# Variable responses to top-down and bottom-up control on multiple traits in the foundational plant, *Spartina alterniflora*

**DOI:** 10.1371/journal.pone.0286327

**Published:** 2023-05-25

**Authors:** Stephanie R. Valdez, Pedro Daleo, David S. DeLaMater, Brian R. Silliman

**Affiliations:** 1 Division of Marine Science and Conservation, Nicholas School of the Environment, Duke University, Beaufort, North Carolina, United States of America; 2 Instituto de Investigaciones Marinas y Costeras (IIMyC), CONICET – UNMDP, Mar del Plata, Argentina; 3 Nicholas School of the Environment, University Program In Ecology, Duke University, Durham, North Carolina, United States of America; Feroze Gandhi Degree College, INDIA

## Abstract

While the effects of top-down and bottom-up forces on aboveground plant growth have been extensively examined, less is known about the relative impacts of these factors on other aspects of plant life history. In a fully-factorial, field experiment in a salt marsh in Virginia, USA, we manipulated grazing intensity (top-down) and nutrient availability (bottom-up) and measured the response in a suite of traits for smooth cordgrass (*Spartina alterniflora*). The data presented within this manuscript are unpublished, original data that were collected from the same experiment presented in Silliman and Zieman 2001. Three categories of traits and characteristics were measured: belowground characteristics, litter production, and reproduction, encompassing nine total responses. Of the nine response variables measured, eight were affected by treatments. Six response variables showed main effects of grazing and/ or fertilization, while three showed interactive effects. In general, fertilization led to increased cordgrass belowground biomass and reproduction, the former of which conflicts with predictions based on resource competition theory. Higher grazing intensity had negative impacts on both belowground biomass and reproduction. This result contrasts with past studies in this system that concluded grazer impacts are likely relegated to aboveground plant growth. In addition, grazers and fertilization interacted to alter litter production so that litter production disproportionately increased with fertilization when grazers were present. Our results revealed both predicted and unexpected effects of grazing and nutrient availability on understudied traits in a foundational plant and that these results were not fully predictable from understanding the impacts on aboveground biomass alone. Since these diverse traits link to diverse ecosystem functions, such as carbon burial, nutrient cycling, and ecosystem expansion, developing future studies to explore multiple trait responses and synthesizing the ecological knowledge on top-down and bottom-up forces with trait-based methodologies may provide a promising path forward in predicting variability in ecosystem function.

## Introduction

Despite a general acknowledgment that both top-down (i.e, consumers) and bottom-up (i.e., resources) drivers can simultaneously regulate plant communities, it is unclear how the relative importance of these factors varies across different plant traits [1, 2]. Most work exploring the effects of bottom-up and top-down forces in simultaneously regulating plant traits has focused primarily on changes in standing aboveground plant biomass [2], largely ignoring changes in other traits [3, 4]. However, both herbivory [5] and resource availability [6, 7] have been shown to induce dramatic variation in traits other than aboveground biomass. The variance in these traits is often unrelated to variance in aboveground biomass, such that measuring aboveground biomass alone does not sufficiently characterize plant response to top-down or bottom-up forcing or indicate plant health [8]. Furthermore, these changes in trait values, while unrelated to aboveground biomass, have still been found to have profound effects on ecosystem function [9–12]. Thus, by limiting observations to changes in aboveground biomass, we constrain our ability to predict possible responses to climate change and other anthropogenic stressors.

Ecological studies in salt marshes formed the basis of the bottom-up paradigm of coastal wetland ecology [13–17]. However, it has since become clear that top-down forces also play a significant role in governing marsh plant biomass and are common in marshes around the world [18–21]. Despite the important role salt marshes play in coastal ecosystems, the mechanisms governing their structure and function remain largely ambiguous in relation to top-down vs. bottom-up effects. This is especially true regarding plant traits, other than aboveground biomass.

The provisioning of salt marsh ecosystem services, such as shoreline protection from erosion [16, 22] and carbon sequestration [12], depend not only on the high productivity of aboveground biomass, but also on other inherent properties that are increasingly threatened by pervasive anthropogenic stressors such as nutrient enrichment and altered trophic structures [23]. Decreases in root-to-shoot biomass allocation ratio, caused by eutrophication or heavy oiling, for example, drive decreases in geomorphic stability, which increases rates of coastal erosion [4, 24]. Salt marshes are also recognized as extremely valuable carbon sinks [12], which depends, in large part, on living roots and soil litter inputs [11]. Furthermore, seed production, through flowering, can have important consequences not only for granivorous species of birds and rodents [25, 26] but also for bare patch dynamics [27], and thus, system resilience against disturbance. Additionally, changes in the production of new stems can affect stand stem density, thereby affecting water flow, sediment stability and retention, evaporation, and soil salinity, as well as refugia availability [28, 29]. Despite the evident importance of other traits beyond aboveground biomass, only a small proportion of marsh studies focus on top-down and bottom-up effects on other plant traits. For example, a comprehensive meta-analysis that synthesized trends in nutrient effects on plant-herbivore interactions in salt marshes and mangroves showed that 68 out of 80 studies focused on standing aboveground biomass while only 9 focused on belowground biomass (S1 Table in S1 File).

Here, we focus on the relative impacts of top-down and bottom-up drivers on other aspects of plant life history, including belowground characteristics, reproduction, and litter production, using the dominant salt marsh plant—smooth cordgrass (*Spartina alterniflora*) as a model species. Employing a manipulative field experiment, we hypothesized an array of interactions among top-down (i.e. snail grazers) and bottom-up (i.e. nutrient availability) factors and their impact on some often overlooked plant traits in salt marsh ecology. We predicted that with increased nutrient availability there would be increased cordgrass reproduction and litter production due to increased aboveground biomass [18], while belowground biomass would decrease with increased nutrient availability [4, 30, 31]. Comparatively, we predicted that grazer pressure would decrease cordgrass reproductive outputs and litter production while increasing belowground biomass by potentially shifting growth allocation from leaf tissues to root structures [32]. If there were a change to resource allocation with grazing, there would potentially be an interaction in which grazers mitigate the negative impacts of increased nutrient availability on cordgrass. Given the context of human direct and indirect alteration to nutrient cycles and food webs in estuarine systems, improving our knowledge about the separate and interactive effect of these controls on often overlooked plant traits may simultaneously advance knowledge and allow us to more accurately predict human impacts on natural systems and their functions linked to specific plant traits.

## Methods

### Study Site

**Fig 1:**
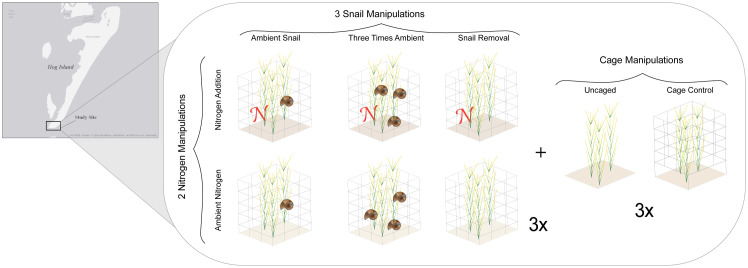
Map and conceptual illustration of experimental design. Map of study site on Hog Island, Virginia, USA and conceptual illustration of experimental design with the following treatments: 1) Nitrogen addition with ambient snails, 2) nitrogen addition with three times ambient snails, 3) nitrogen addition without snails, 4) ambient nitrogen with ambient snails, 5) ambient nitrogen with three times ambient snails, and 6) ambient nitrogen without snails. The figure also depicts cage controls and uncaged plots used to assess caging effects on marsh plants. The map in the figure was created in R using ggmap [33] from ©OpenStreetMap under a ODb license, with permission from OpenStreetMapFoundation, original copyright 2018. https://www.openstreetmap.org/copyright.

A field experiment was carried out on Hog Island, Virginia USA (Fig 1) in the growing season (May through September) of 1997. This island, a barrier island in central Virginia, USA, is part of the Virginia Coast Reserve Long Term Ecological Research (VCR LTER) project. We were granted permission by the Nature Conservancy in collaboration with the VCR to conduct the study within the LTER. While no official permits were required for this study, we followed standard practices and observed the stated laws. Hog Island is a barrier island bordering the Atlantic Ocean on the east and Hog Island Bay on the west. Sediments are characterized as a mix of mud and sand–with sand originating from frequent overwash events on this narrow barrier island. Work was done on the southern portion of the island in the intermediate *S*. *alterniflora* zone, where salt marsh periwinkle snails (*Littorina irrorata*) are abundant. Original methods for the experimental setup are described in Silliman and Zieman 2001 [18]. Here we present original, unpublished data from that 1997 experiment.

### Experimental design

To evaluate the separate and interactive effects of bottom-up (i.e. nutrient availability) and top-down (i.e.grazing pressure) forces, we implemented a 2x3 factorial experiment manipulating the availability of the main limiting nutrient (i.e. nitrogen [34]) to marsh plant growth and the abundance of an important grazer (i.e. the marsh periwinkle; [18]) in the system (Fig 1). The experiment was initiated on 5 May 1997 and monitored until the first week of September 1997. Two levels of nitrogen availability (ambient and fertilized) and three levels of grazer manipulation were applied (ambient snail abundance, three times ambient snail abundance, and snail removal). The resulting six treatments are referred to as: 1) snail removal, 2) snail control, 3) snail addition, 4) snail removal + nitrogen, 5) snail control + nitrogen, and 6) snail addition + nitrogen (Fig 1, n = 3). We measured the response of these treatments on nine traits and/or characteristics that we categorized as follows: 1) belowground characteristics include belowground biomass (g/m^2^), the ratio of belowground to aboveground biomass (g/m^2^), and organic soil content (% organic matter), 2) litter production includes standing dead mass (dry mass g/m^2^) and leaf litter (dry mass g/m^2^), and 3) reproduction includes the number of flowering shoots/ m^2^, proportion of total shoots that are flowering, number of new shoots/ m^2^, and mean length of new shoots from sediment (cm). All plots were enclosed within 1 m^2^ roofless cages constructed of 12.7 mm galvanized hardware mesh 76.2 cm high. Two additional cages were constructed for each treatment for destructive sampling. Cages were extended 5 cm into the sediment to prevent snail movement, and plant root systems were cut up to 40 cm depth to prevent resource sharing outside of the plots. All plots were placed at the same elevation to control for water inundation. A wooden boardwalk was created to walk between plots without sediment disruption.

Caging effects, if any, were assessed from uncaged plots and control cages (n = 3). Cage control plots were implemented using cages of the same dimensions as experimental plots, but were not manipulated for snails and nitrogen fertilization, and cages were lifted off the sediment, leaving a small gap to allow snail movement between the plot and the surrounding area. Uncaged plots were plots of the same area without any caging material. The same sampling procedure for treatment plots occurred in the three uncaged plots and three caged controls for belowground biomass, number of flowering shoots, number of new shoots, and length of new shoots.

### Nitrogen enrichment

Nitrogen, as ammonium chloride (NH_4_Cl), was added to the sediment via 16 evenly spaced 15mL centrifuge tubes filled with 3.4g of NH_4_Cl wrapped in nylon mesh to promote slow release [35]. Enough NH_4_Cl was added to nitrogen fertilized plots to theoretically double the production of *S*. *alterniflora* (projected from [34]; 1996 *S*. *alterniflora* aboveground production at this site = 425 g dry mass m^-2^ yr ^-1^). No fertilizer was added to ambient nitrogen plots. Empty tubes were inserted into non-fertilized plots as disturbance controls for the added structure. At the conclusion of the experiment, organic soil content was measured by extracting a 1mL round core from the surface using a 5 ml syringe that was rinsed with seawater between samples. Organic content was determined by weight from loss of ignition at 450°C.

### Grazer densities

Naturally occurring snail densities were determined at the start and conclusion of the experiment by counting snails in 75 randomly placed 1 m^2^ quadrats in stands of intermediate height form *S*. *alterniflora*. At the beginning of the experiment, snails were removed from all enclosures. Snails were reintroduced by pre-determined grazer levels in snail control and snail addition treatments. Control snail treatments had naturally occurring densities (48 snails/ m^2^) while snail addition treatments had three times the amount of snail control treatments (144 snails/ m^2^), a naturally occurring high snail density for mid-Atlantic marshes [36, 37].

Snail densities were monitored weekly, and snails were added or removed if they strayed from intended levels. Average snail shell height of 45 randomly selected snails was calculated every two weeks to account for variation in snail size. Furthermore, due to the enhanced growth of smooth cordgrass in the presence of the Atlantic marsh fiddler crabs (*Uca pugnax*) and the ribbed mussels (*Geukensia demissa)*, density of these organisms was also assessed every two weeks [38, 39]. The purple marsh crab (*Sesarma reticulatum*), another grazer of *S*. *alterniflora*, was excluded by cage treatments and no burrows were observed within plots. No differences in mussel or fiddler crab densities were observed between treatments [18].

### Plant measurements

At the conclusion of the experiment, sediment cores (15 cm diameter and 30 cm deep) were taken from each plot to measure plant biomass. The cores were taken to 30 cm depth as that is the approximate extent to which *S*. *alterniflora* extends its roots and root growth takes place in the intermediate marsh zone [40]. The above- and belowground portions of the plants were separated, dried to a constant weight, and reported as dry mass (g)/ m^2^. Aboveground biomass data (reported in [18]) was used to calculate the ratio of belowground to aboveground biomass. Leaf litter was collected from the marsh surface of 0.25m^2^ corner of the 1m^2^ plots. Standing dead mass was removed from the entirety of the plots. Both leaf litter and standing dead mass were dried, weighed, and reported as mass (g/m^2^). The effectiveness of snail manipulation treatments on the abundance and length of grazing scars known as radulations on live *S*. *alterniflora*, was measured previously [18]. In short, there were no radulations within total grazer exclosure treatments and nearly double the radulations marks in addition treatments, showing that grazer exclusion and additions were effective in manipulating grazer pressure [18].

Flowering *S*. *alterniflora* shoots per m^2^ were counted in September 1997 (i.e. 4 months after treatments began). The proportion of flowering shoots was calculated from the total shoot density, also taken in September. The abundance of new shoots was counted from a 0.25 m^2^ quadrat within plots in the first week of August 1997, and again in the first week of September 1997 based on height differential from adult plants. The length (cm) of each new shoot was measured from the sediment to the tip of the longest blade and the mean was derived for each treatment.

### Statistical analysis

All analyses were conducted in R version 4.1.1 [41]. Potential effects of caging were assessed for belowground biomass, proportion of flowering shoots, and number of new shoots, using t-tests between uncaged and caged control plots.

We assessed the effects of treatments on belowground biomass (g/m^2^), ratio of belowground to aboveground biomass (g/m^2^), radulations (cm/stem), leaf litter (g/m^2^), standing dead mass (g/m^2^), organic soil content (% organic content), number flowering, and proportion of flowering shoots using two-way Analysis of Variance (ANOVA) followed by Tukey’s post-hoc test for each dependent variable separately. In all cases, the degrees of freedom for the main effects of nitrogen level, grazer level, and nitrogen by grazer interaction were 1, 2, and 2, respectively. ANOVA model assumptions were evaluated and met in all cases.

Additionally, for the number of new shoots and mean length of new shoots, two-way, repeated measures ANOVAs were used with the “ez” package in R [42]. Neither repeated measure ANOVA resulted in significant snail, nitrogen, and time interactions. Therefore, we conducted two-way ANOVAs evaluating the treatment effects of nitrogen and snail manipulation for each month (August and September) separately.

## Results

**Fig 2:**
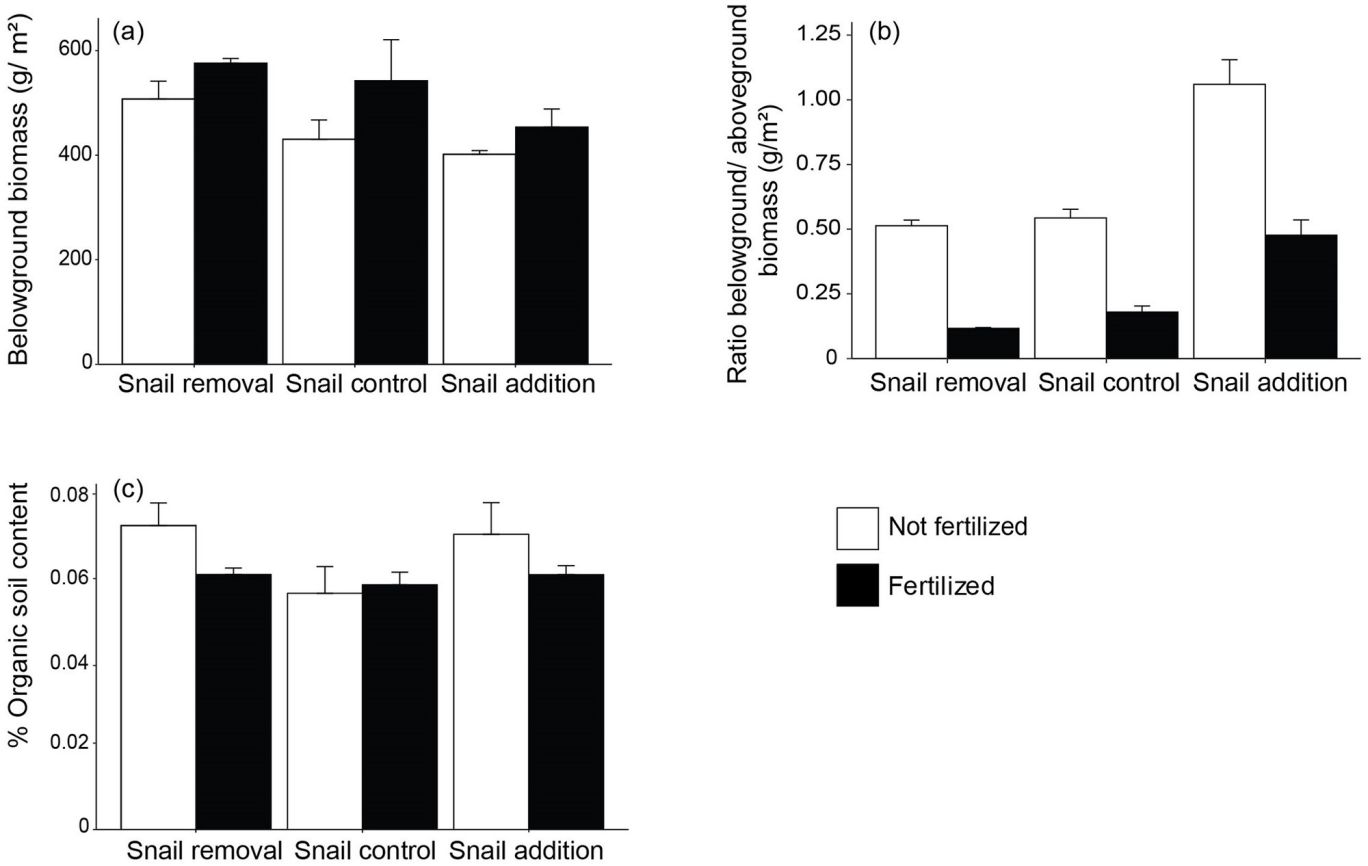
Effects of fertilization and snail (*Littorina irrorata*) density manipulation on belowground characteristics. (A) *Spartina alterniflora* belowground biomass (g/m^2^), (B) Ratio of belowground to aboveground biomass of *S*. *alterniflora* (g/m^2^) and (C) % organic soil content. Results of two-way ANOVAs given, testing the main and interactive effects of grazers (G) and fertilization (N). Error bars represent standard error (n = 3).

**Fig 3:**
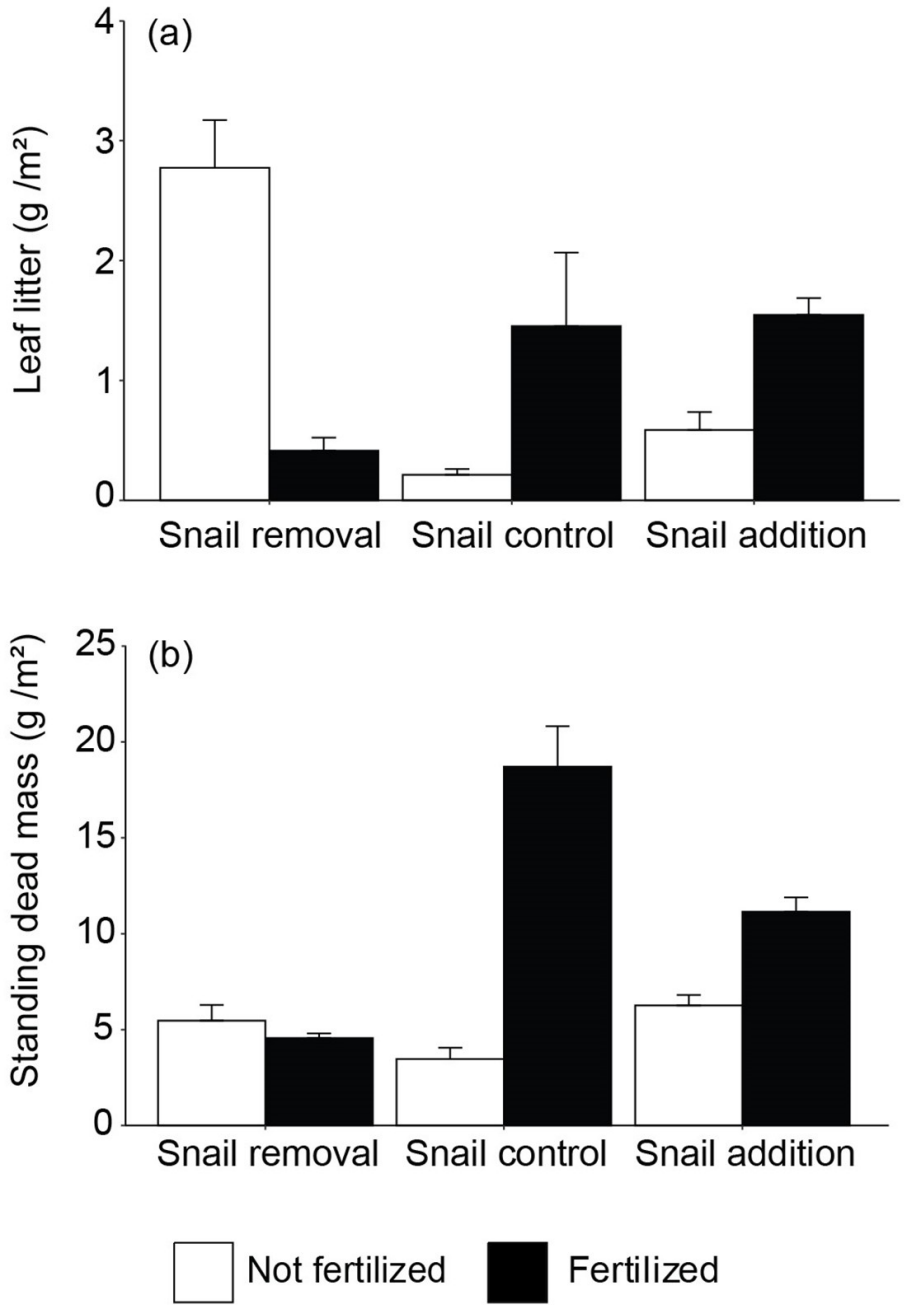
Effects of fertilization and snail (*Littorina irrorata*) density manipulation on litter production. (A) aboveground standing dead mass (dry weight (g)/m^2^) and (B) leaf litter (dry weight (g)/m^2^). Error bars represent standard error (n = 3).

We did not detect differences between cage controls and uncaged plots in the six variables examined (S2 Table, S1 and S2 Figs in S1 File). Independent effects of both nitrogen availability and grazer density were detected for belowground biomass where belowground biomass increased by 26% with fertilization (*F*_1,12_ = 5.4, *P* = 0.039) and decreased by 26.3% between snail removal and snail addition (*F*_2,12_ = 3.9, *P* = 0.0494; Fig 2A, Table 1, see also S3 Table in S1 File). However, the ratio between belowground and aboveground biomass decreased with fertilization by 66.8% (*F*_1,12_ = 122.9, *P*< 0.0001) and increased with snail density by 51.6% from snail removal to snail addition (*F*_1,12_ = 50.8, *P*< 0.0001; Fig 2B, Table 1, see also S3 Table in S1 File). Organic soil content was not influenced by either fertilization or snail density (Fig 2C, Table 1). Traits related to litter production demonstrated interactive effects of fertilization and snail density (leaf litter: *F*_2,12_ = 20.3, *P* = 0.0001; standing dead mass: *F*_2,12_ = 31.42, *P*> 0.0001; Fig 3, Table 1, see also S3 Table in S1 File). Here, leaf litter increased by 274.2% between fertilized plots without versus those with grazer additions but decreased under ambient nutrient conditions by 78.9% from no grazer to grazer additions. Standing dead mass peaked by 3-fold in fertilized plots with ambient grazer density compared to fertilization without grazers.

**Table 1:** Reported mean values and corresponding ANOVA significance.

**A) Means**	**Measured Traits**					
**Nitrogen *Grazer Level***	**Belowground Biomass (g/m2) ± SE**	**Ratio Below: Aboveground Biomass (g/m2) ± SE**	**Radulations (cm/ Stem) ± SE**	**Organic Soil Content± SE**	**Standing Dead (dry mass g/ m2) ± SE**	**Leaf Litter (dry mass g/ m2) ± SE**
Ambient						
*Removal*	507.3 ± 34.0	0.513 ± 0.02	0.003 ± 0.002	0.070 ± 0.005	5.47 ± 0.82	2.78 ± 0.40
*Control*	430.3 ± 36.6	0.543 ± 0.03	0.40 ± 0.14	0.055 ± 0.006	3.47 ± 0.58	0.21 ± 0.05
*Addition*	401.6 ± 6.86	1.06 ± 0.10	0.80 ± 0.34	0.068 ± 0.007	6.26 ± 0.54	0.59 ± 0.15
Fertilized						
*Removal*	575.7 ± 8.85	0.117 ± 0.003	0.008 ±0.003	0.059 ± 0.002	4.56 ± 0.25	0.41 ± 0.11
*Control*	542.2 ± 78.4	0.180 ± 0.02	0.93 ±0.39	0.057 ± 0.003	18.71 ± 2.12	1.45 ± 0.62
*Addition*	453.3 ± 35.0	0.477 ± 0.06	1.42 ± 0.61	0.059 ± 0.002	11.14 ± 0.75	1.55 ± 0.24
	**Number**	**Proportion**	**August New**	**September**	**August Mean Length of New Shoots (cm)** ± **SE**	**September Mean Length of New Shoots (cm)** ± **SE**
**Nitrogen *Grazer Level***	**Flowering/ m2 ± SE**	**Flowering /m2 ± SE**	**Shoots/ m2 ± SE**	**New Shoots/ m2 ± SE**		
Ambient						
*Removal*	25.33 ± 1.76	10.56 ± 0.23	218.67 ± 14.11	458.67± 103.83	5.98± 0.33	8.14± 0.60
*Control*	10.0 ± 1.53	4.92 ± 0.67	144.0 ± 9.23	277.33± 61.51	4.37± 0.73	7.45± 1.45
*Addition*	2.67 ± 0.88	1.68 ± 0.41	74.67 ± 14.11	197.33± 37.33	4.90± 0.33	6.73± 0.14
Fertilized						
*Removal*	56.0 ± 6.93	17.4 ± 2.88	384.0 ± 64.66	506.67± 52.53	8.66± 0.23	9.11± 0.76
*Control*	20.67 ± 5.21	7.91± 2.88	181.33 ± 29.69	373.33± 74.09	8.36± 1.54	12.31± 0.33
*Addition*	6.33 ± 2.73	3.69 ± 2.62	292.33 ± 61.51	325.33± 65.54	7.32± 0.18	10.99± 1.28
**B) ANOVA**	**Below ground Biomass**	**Ratio Below: Aboveground Biomass**	**Radulations**	**Organic Soil Content**	**Standing Dead**	**Leaf Litter**
**Source**						
Nitrogen (N)	*	***	NS	NS	***	NS
Grazer Level (G)	*	***	*	NS	**	NS
N + G	NS	NS	NS	NS	***	**
**Source**	**Number Flowering**	**Proportion Flowering**	**August New Shoots**	**September New Shoots**	**August Mean Length of New Shoots**	**September Mean Length of New Shoots**
Nitrogen (N)	***	**	**	NS	***	**
Grazer Level (G)	***	***	*	*	NS	NS
N + G	*	NS	NS	NS	NS	NS

(A) Mean responses to snail (*Littorina irrorata*) density and nitrogen manipulation on *Spartina alterniflora* and (B) corresponding ANOVA significance where *P < 0.05; **P < 0.01; ***P < 0.001; NS, not significant (P > 0.05)

**Fig 4:**
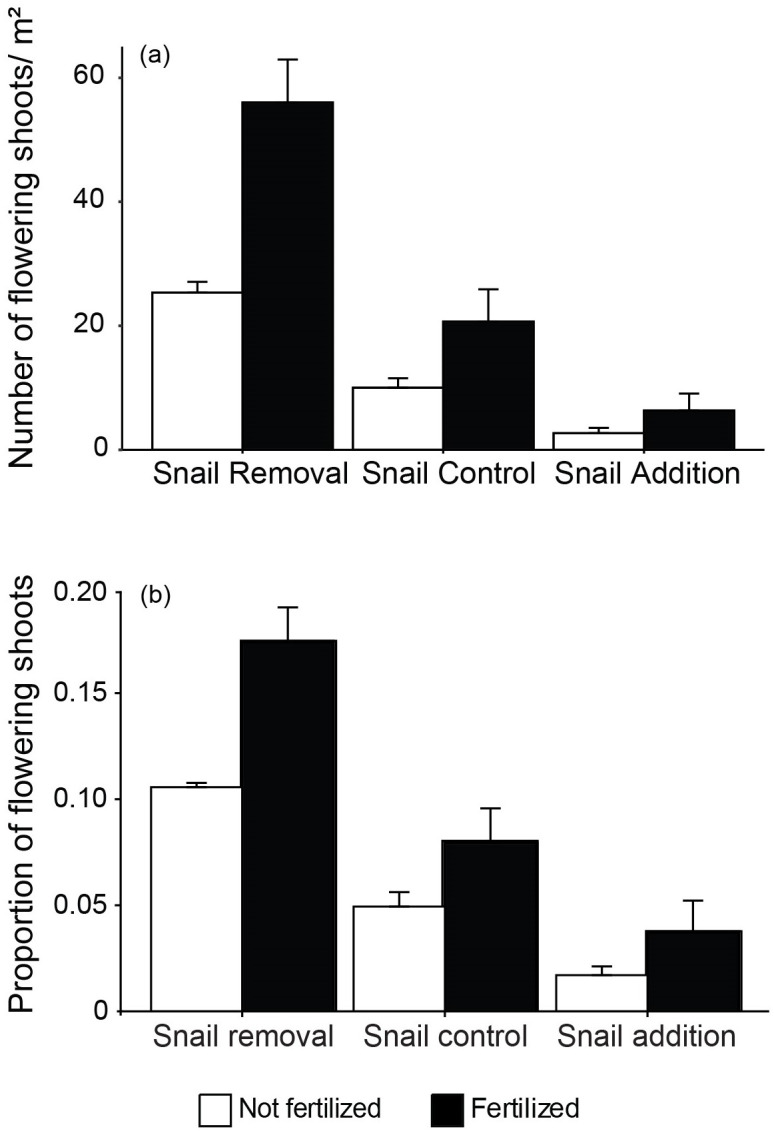
Effects of fertilization and snail (*Littorina irrorata*) density manipulation on sexual reproduction. (A) number flower/ m^2^ and (B) proportion of flowering shoots. Error bars represent standard error (n = 3).

**Fig 5:**
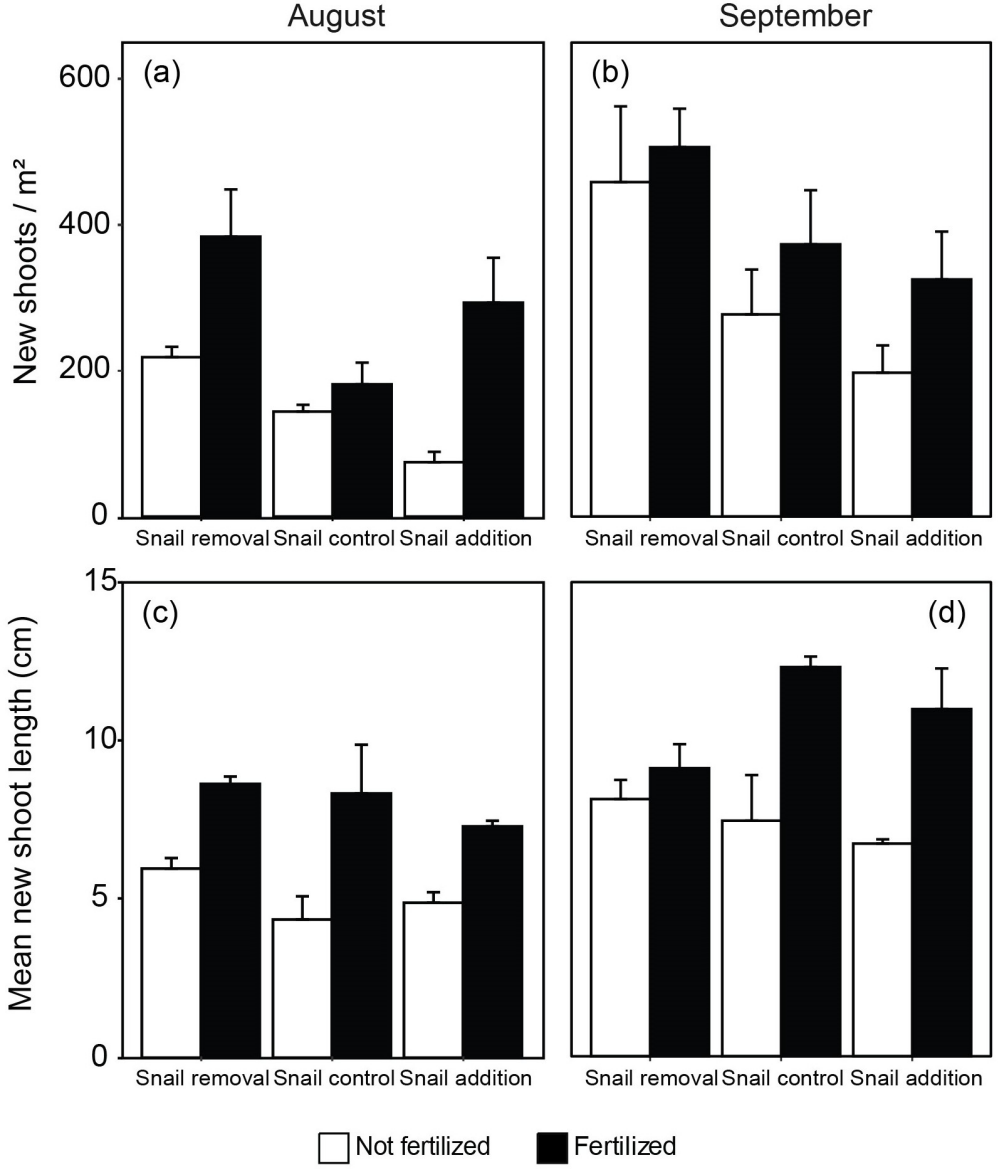
Effects of fertilization and snail (*Littorina irrorata*) density manipulation on asexual reproduction. The number of new shoots in August (A) and September (B), and length (cm) of new shoots in August (C) and September (D) under nitrogen and snail (*Littorina irrorata*) density manipulations. Error bars represent standard error (n = 3).

Sexual reproduction traits had varying impacts of fertilization and grazer density. The number of flowering shoots demonstrated interactive effects of fertilization and grazer density (*F*_2,12_ = 6.64, *P* = 0.011) in which flower density increased with fertilization and decreased with greater grazer density (Fig 4A, Table 1, see also S3 Table in S1 File). There was an 88.6% decrease in flowering between fertilized plots with no grazers and fertilized plots with grazer addition. The proportion of flowering shoots had similar trends with fertilization (60.2% increase in flowering in fertilized versus unfertilized treatments) and grazer density (83.9% decrease in flowering in no grazer versus grazer addition treatments) but no interaction (fertilization; *F*_1,14_ = 16.71, *P*< 0.0015, snail density; *F*_2,14_ = 47.3, *P*< 0.0001) (Fig 4B, Table 1, see also S3 Table in S1 File). Finally, asexual reproductive traits revealed impacts of fertilization and grazer density, although not consistent through time. The number of new shoots in August increased by 25.9% with fertilization (*F*_1,14_ = 19.03, *P* = 0.0009) and decreased by 65.9% with snail density (*F*_2,14_ = 7.17, *P* = 0.009) (Fig 5A and 5C, Table 1, see also S3 Table in S1 File). The length of new shoots in August only increased with fertilization by 91.1% between unfertilized and fertilized treatments without grazers (*F*_1,14_ = 25.6, P = 0.0003). In September, the number of new shoots decreased with grazer density by 56.9% between no grazer and grazer additions in unfertilized treatments (*F*_2,14_ = 5.46, *P* = 0.02) and no effect of fertilization while the length of new shoots increased by 65.2% with fertilization (*F*_1,14_ = 21.2, *P* = 0.0006) and no effect of grazer density (Fig 5B and 5D, Table 1, see also S3 Table in S1 File).

## Discussion

Our results show that both top-down and bottom-up forces impact a suite of traits beyond the commonly measured aboveground responses. All three classes of traits measured (belowground characteristics, litter production, and reproduction) demonstrated effects of increased nutrient availability and grazer density. However, the occurrence, direction, and size of these effects were not consistent across traits, including aboveground biomass. Three of the nine traits measured demonstrated an interaction between fertilization and grazing: leaf litter, standing dead mass, and number of flowering shoots. Of the six remaining traits measured, one showed no impact of either grazing or fertilization: organic soil content. The other five responses measured were affected by fertilization, grazing, or both as main effects.

Belowground characteristics, such as the root structure and biomass, are critical in determining the stability of marshes in the face of rising seas [30] and in carbon storage [43]. Here, as nitrogen availability increased, so did belowground biomass (Fig 2). This is in contrast with our predictions and with other experimental findings that have found decreases in belowground biomass with fertilization [4, 30, 31]. This discrepancy may be due to the disparate locations in the marsh (high vs. low marsh) in which the studies took place. An increase in belowground structure could lead to more stable substrate, thereby improving erosion protection and increased carbon storage through sediment accretion and burial of belowground plant material [8, 44–46]. However, and potentially more ecologically significant, the ratio between belowground to aboveground biomass decreased with nitrogen additions due to a larger increase in aboveground biomass [18], which supports what is commonly found across wetland plant species (Fig 3) [47, 48]. Although both above and belowground biomass increase, the shift to relatively dominant aboveground structure could have consequences for marsh functioning, stability, and may negate some of the positive impacts mentioned above generated by increased belowground structures. As the ratio between above and belowground biomass becomes more disparate, the resisting forces to erosion and sediment loss provided by belowground biomass may be exceeded by eroding forces such as hydrodynamic action on aboveground biomass [22]. This could create a net negative impact on cordgrass, even though both above and belowground biomass increase with nutrient availability. For example, eutrophication via agricultural run-off has generally resulted in diminished biomass accumulation over the long-term, leading to potential changes in ecosystem services [30, 31] and resilience in salt marshes to edge erosion and collapse [4]. Future research is needed to resolve when and where belowground biomass in marsh plants either positively or negatively responds to nitrogen enrichment and this work should be conducted across marsh plant zones.

Grazing also had effects on both belowground biomass and the ratio between below- and aboveground biomass. As grazing pressure increased, belowground biomass decreased while the ratio between below and aboveground biomass increased. Our results combined with those previously presented [18] show a dramatic decrease in aboveground biomass, demonstrate a holistic change to plant structure in a single growing season. This likely occurred because of the strong top-down effect of snails on aboveground structures which then likely lead to increased demand for resources from belowground storage structures. Studies in grasslands suggest that the loss of aboveground tissue to grazing results in a change in resource allocation from belowground root structures to aboveground leaf tissue to compensate for the loss [32]. Further studies are needed to better understand the relationship and potential compensation between below and aboveground biomass with grazing in salt marshes.

Fertilization and grazing interactively affected litter production (i.e. standing dead mass and leaf litter, Fig 3), where dead material generally increased with fertilization, especially with grazers present, which is a similar pattern seen in standing live aboveground biomass [18]. This pattern suggests a shift in grazing pressure so that snail impact on live grass increases with increasing nitrogen availability. This possibility is supported by published results of this experiment [18] that show increased per capita snail grazing on fertilized plants. Periwinkle snails have been considered part of the brown food web, largely consuming detrital material. However, research has found that periwinkle snails engage in fungal farming behavior by grazing live leaf tissue and facilitating fungal growth by depositing spore-laden feces into the grazing wounds [49]. The increase in aboveground, nutrient-rich biomass with fertilization could shift snail feeding preference from standing dead detrital material to fungal farming on live grass [50], allowing an accumulation of leaf litter and increasing plant susceptibility to disease, stress, and die off, resulting in more standing dead mass [49, 51]. The likely fate of this increased production of dead plant material will either become part of the detrital food web (*in situ* consumption of dead material by marsh invertebrates), be washed out of the system, or become buried carbon that contributes to a long-term carbon pool [12, 52, 53]. Detrital material plays an important role in marsh ecosystem functioning and nutrient recycling, including supporting higher trophic levels [54] Additionally, an expanded brown food web caused by increased detrital production can extend to other ecosystems post-consumption through the transportation of organisms, thereby supporting adjacent food webs such as deeper water fish communities [55, 56]. Therefore, the impacts of increased detrital production may be far-reaching and diverse in ecosystem response. Comparatively, increased dead plant material contribution to a carbon pool may establish long-term changes to carbon storage with increased nutrient loads that may be coupled with changes to belowground biomass [4, 57]. The interacting impacts of grazers and nutrients on detrital material are likely far reaching and complicated but are valuable to ecosystem functioning and require more investigation.

Grazing and fertilization had mixed effects on cordgrass reproduction, both sexual and asexual. The number of flowering shoots was the other trait (besides litter production traits) that exhibited an interaction between grazing and nutrient enrichment (Fig 4). In general, fertilization increased the number of flowering shoots. However, at high levels of snail grazers, the number of flowering shoots was diminished with and without fertilization. This could suggest a shift in plant growth allocation away from reproduction to self-maintenance under grazer pressure, regardless of fertilization [58]. Fertilization increased both the proportion of flowering shoots, the number of new shoots, and the mean length of new shoots (Figs 4 and 5). The increase in aboveground adult plant biomass with fertilization, previously described [18], combined with our findings of the greater number of new shoots, increased length of new shoots, and increased proportion of flowering shoots with fertilization, suggests a cumulative increase in areal coverage of cordgrass and an increased investment in sexual reproduction following increased nitrogen availability. The combined effects of more aboveground biomass and flowering are consistent with previous results that suggest cordgrass is more likely to flower with greater biomass and height [59]. The increase in both the proportion of flowering and new shoots could lead to a strengthening of existing ecosystem services salt marshes provide, as well as contribute to and expand local salt marsh systems. For example, the increase in new shoots indicates increased primary productivity and is tied to trophic transfer through the food web, carbon sequestration rates, and sedimentation that may provide resilience to sea level rise [60–62]. Alternatively, increased grazing pressure either reduced or had no effect on cordgrass asexual reproduction (abundance and length of new shoots) while it decreased sexual reproduction (flowering). For example, grazing pressure did not influence the length of new shoots. This suggests that grazers may either focus efforts on older individuals, not impacting new shoot lengths, or new shoots that do experience grazing may not frequently survive which is supported by the decrease abundance of new shoots with increased grazing pressure. Additionally, these results could have been observed because increased grazing decreases belowground biomass creating a deficit in energy available to support new shoots through the initial growth stages. Young plants are dependent on carbon storage and are not yet a carbon source for the greater genet. Additionally, the proportion of flowering shoots decreased with increased grazers. Cordgrass mostly depends on vegetative reproduction, given the low seed viability (only lasting a year) and the lack of reliable seed banks on salt marshes [63]. The reliance on vegetative reproduction may be detrimental to long-term survival as marshes face many anthropogenic stressors. A decrease in overall reproductive output could diminish ecosystem function and services provided by the marsh as well as an increase in marsh susceptibility to global change stressors.

Periwinkles, the grazing snail used in this study, have been shown to dramatically reduce the biomass of cordgrass, creating large die-off areas, lowering the plant’s ability to withstand environmental stress, and diminishing associated ecosystem services [50, 51, 52, 64, 65]. Researchers found that this diminution is not a linear function of area loss, as up to half of salt marsh sediment storage ability is lost when a quarter of the marsh dies off [66]. The continuum of positive and negative impacts of grazing and fertilization across traits demonstrates the converging, and often conflicting, forces of top-down and bottom-up effects on the structure and function of primary foundation species. Furthermore, nutrient enrichment greatly increased herbivory in salt marshes. In this way, these opposing top-down and bottom-up forces could create a negative feedback loop, which would dampen and stabilize any effects presented on their own [51]. However, other combinations of top-down and bottom-up effects might be more detrimental in certain environmental contexts. For example, drought decreases cordgrass growth and its ability to resist herbivores, which results in mass die-offs of cordgrass and a collapse of these systems [52] which may be exacerbated under high nutrient loads. For now, the interactive effects of top-down and bottom-up forces are largely suggestive. Therefore, more spatially diverse and highly replicated research is needed to fully understand these interacting forces on many traits.

While the knowledge of herbivory and fertilization as oppositional top-down and bottom-up forces on a suite of cordgrass traits is useful to elevate our understanding of how these forces contribute to the structure and function of natural systems, it also provides knowledge which might be used to better manage salt marsh systems. By understanding the effects that elevated herbivory or fertilization might have on more than one cordgrass trait, we could also better predict impacts of local and global changes on marshes. These predictions and management strategies can be better informed using a compilation of interacting traits, rather than the limited response to aboveground biomass, which, as we have demonstrated, does not depict the full impacts of changing nutrient availability and grazer pressure. While this is useful knowledge to anticipate effects on marsh habitat, the full picture of what forces regulate salt marsh habitats is undoubtedly far more complicated than the two oppositional forces of herbivory and fertilization.

## Supporting information

S1 File (DOCX)
