# Supplementary material for: Variable responses to top-down and bottom-up control on multiple traits in the foundational plant, Spartina alterniflora
Source: PLoS One. 2023 May 25;18(5):e0286327. doi: 10.1371/journal.pone.0286327 (PMC10212092; doi:10.1371/journal.pone.0286327)
Supplement: S1 File — (DOCX) [file pone.0286327.s001.docx]

**Top-down and bottom-up control on multiple traits in a foundational plant**

Stephanie R. Valdez, Pedro Daleo, David S. DeLaMater III, Brian R. Silliman

**Supplemental Material:**


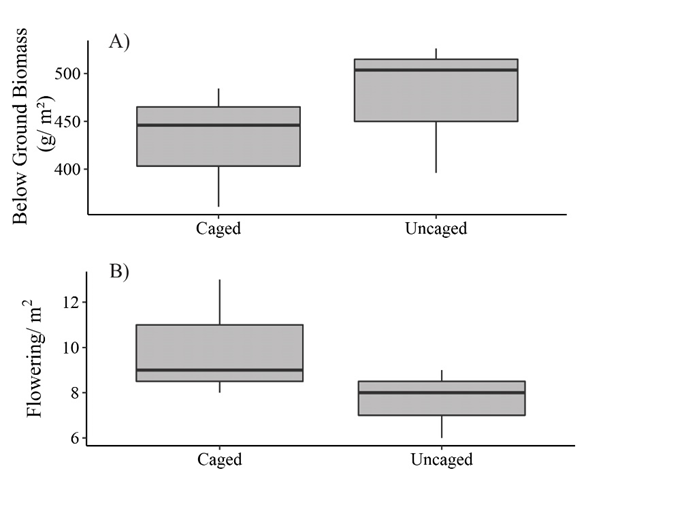


**S1 Figure:** **Graphical comparison of caged and uncaged** **plots**

A) belowground biomass (g/m^2^) and B) number of flowering shoots/ m^2^.


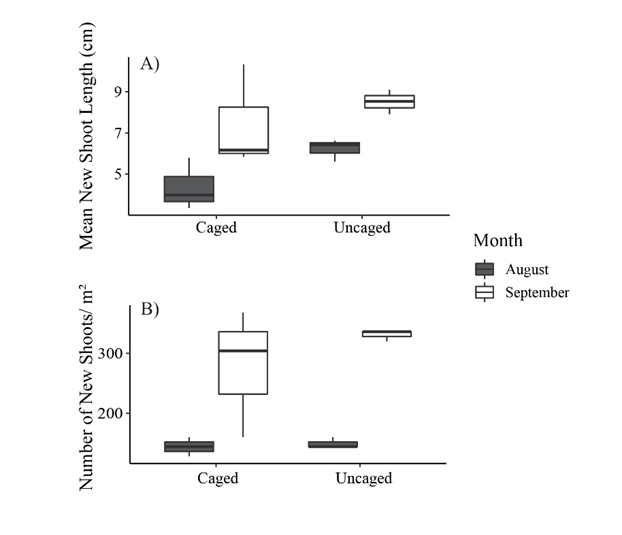


**S2 Figure:** **Graphical comparison of cages and uncaged plots**.

A) mean length of new shoots (cm) and B) number of new shoots/ m^2^ in August and September

**S1 Table:** **Literature review of studies that explored traits beyond aboveground biomass.**

| **Code** | **Aboveground biomass** | **Belowground biomass** | **Others** |
| --- | --- | --- | --- |
| **A001** | yes |  |  |
| **A002** | yes | yes |  |
| **A003** | yes |  |  |
| **A004** | yes |  |  |
| **A005** |  |  | Plant nutrient parameters |
| **A006** | yes |  |  |
| **A007** | yes |  |  |
| **A008** | yes |  |  |
| **A009** | yes |  |  |
| **A010** | yes |  |  |
| **A011** | yes | yes |  |
| **A012** | yes |  |  |
| **A013** |  |  | Plant nutrient parameters |
| **A014** | yes |  |  |
| **A015** | yes |  |  |
| **A016** | yes |  |  |
| **A017** |  |  | Plant nutrient parameters |
| **A018** | yes |  |  |
| **A019** | yes |  |  |
| **A020** |  |  | Plant nutrient parameters |
| **A021** | yes |  |  |
| **A022** | yes | yes |  |
| **A023** | yes |  |  |
| **A024** | yes |  |  |
| **A025** | yes |  |  |
| **A026** |  |  | Plant nutrient parameters |
| **A027** |  |  |  |
| **A028** | yes |  |  |
| **A029** |  |  | Insect survival, abundance or biomass |
| **A030** | yes |  |  |
| **A031** | yes |  |  |
| **A032** | yes |  |  |
| **A033** | yes |  |  |
| **A034** |  |  | Insect survival, abundance or biomass |
| **A035** | yes |  |  |
| **A036** | yes |  |  |
| **A037** | yes |  |  |
| **A038** | yes |  |  |
| **A039** |  |  | Insect survival, abundance or biomass |
| **A040** | yes |  |  |
| **A041** | yes | yes |  |
| **A042** | yes |  |  |
| **A043** | yes |  |  |
| **A044** | yes |  |  |
| **A045** |  |  | Gall density, plant nutrient parameters |
| **A046** |  |  | Gall density, plant nutrient parameters |
| **A047** | yes |  |  |
| **A048** |  |  | Gall density, plant nutrient parameters |
| **A049** | yes | yes |  |
| **A050** | yes |  |  |
| **A051** |  |  | Hydraulic connectivity |
| **A052** | yes |  |  |
| **A053** | yes |  |  |
| **A054** |  |  | Leaf damage by herbivores |
| **A055** | yes |  |  |
| **A056** | yes |  |  |
| **A057** | yes | yes |  |
| **A058** | yes |  |  |
| **A059** | yes |  |  |
| **A060** | yes |  |  |
| **A061** | yes |  |  |
| **A062** | yes |  |  |
| **A063** | yes |  |  |
| **A064** | yes | yes |  |
| **A065** | yes |  |  |
| **A066** | yes |  |  |
| **A067** | yes |  |  |
| **A068** | yes |  |  |
| **A069** | yes |  |  |
| **A070** | yes |  |  |
| **A071** | yes |  |  |
| **A072** | yes |  |  |
| **A073** | yes |  |  |
| **A074** | yes | yes |  |
| **A075** | yes |  |  |
| **A076** | yes |  |  |
| **A077** |  |  | NA |
| **A078** |  |  | Leaf damage by herbivores |
| **A079** | yes |  |  |
| **A080** | yes | yes |  |

Estimation of the number of studies focused on cordgrass (*Spartina alterniflora*) standing aboveground biomass, on belowground biomass or on other response variables. Used publications are those included in He and Silliman (2015), see below for the full citations associated with the codes.

A001. Jefferies, R.L. & Perkins, N. (1977). The effects on the vegetation of the additions of inorganic nutrients to salt marsh soils at Stiffkey, Norfolk. *J. Ecol.*, 65, 867-882.

A002. Haines, E.B. (1979). Growth dynamics of cordgrass, *Spartina alterniflora* Loisel., on control and sewage sludge fertilized plots in a Georgia salt marsh. *Estuaries*, 2, 50-53.

A003. Mendelssohn, I.A. (1979). The influence of nitrogen level, form, and application method on the growth response of *Spartina alterniflora* in North Carolina. *Estuaries*, 2, 106-112.

A004. Covin, J. & Zedler, J. (1988). Nitrogen effects on *Spartina foliosa* and *Salicornia virginica* in the salt marsh at Tijuana Estuary, California. *Wetlands*, 8, 51-65.

A005. Stiling, P., Brodbeck, B.V. & Strong, D.R. (1991). Population increases of planthoppers on fertilized salt-marsh cord grass may be prevented by grasshopper feeding. *Fla. Entomol.*, 74, 88-97.

A006. Clarke, P.J. & Allaway, W.G. (1993). The regeneration niche of the grey mangrove (*Avicennia marina*): effects of salinity, light and sediment factors on establishment, growth and survival in the field. *Oecologia*, 93, 548-556.

A007. Osgood, D.T. & Zieman, J.C. (1993). Factors controlling aboveground *Spartina alterniflora* (smooth cordgrass) tissue element composition and production in different-age barrier island marshes. *Estuaries*, 16, 815-826.

A008. Feller, I.C. (1995). Effects of nutrient enrichment on growth and herbivory of dwarf red mangrove (*Rhizophora mangle*). *Ecol. Monogr.*, 65, 477-505.

A009. Boyer, K.E. & Zedler, J.B. (1996). Damage to cordgrass by scale insects in a constructed salt marsh: Effects of nitrogen additions. *Estuaries*, 19, 1-12.

A010. Dai, T. & Wiegert, R.G. (1996). Ramet population dynamics and net aerial primary productivity of *Spartina alterniflora*. *Ecology*, 77, 276-288.

A011. Koch, M.S. & Snedaker, S.C. (1997). Factors influencing *Rhizophora mangle* L. seedling development in Everglades carbonate soils. *Aquat. Bot.*, 59, 87-98.

A012. Ruess, R.W., Uliassi, D.D., Mulder, C.P.H. & Person, B.T. (1997). Growth responses of *Carex ramenskii* to defoliation, salinity, and nitrogen availability: Implications for geese-ecosystem dynamics in western Alaska. *Ecoscience*, 4, 170-178.

A013. Stiling, P. & Rossi, A.M. (1997). Experimental manipulations of top-down and bottom-up factors in a tri-trophic system. *Ecology*, 78, 1602-1606.

A014. Boyer, K.E. & Zedler, J.B. (1998). Effects of nitrogen additions on the vertical structure of a constructed cordgrass marsh. *Ecol. Appl.*, 8, 692-705.

A015. Levine, J.M., Brewer, J.S. & Bertness, M.D. (1998). Nutrients, competition and plant zonation in a New England salt marsh. *J. Ecol.*, 86, 285-292.

A016. Gough, L. & Grace, J.B. (1998). Effects of environmental change on plant species density: Comparing predictions with experiments. *Ecology*, 80, 882-890.

A017. Rossi, A.M. & Stiling, P. (1998). The interactions of plant clone and abiotic factors on a gall-making midge. *Oecologia*, 116, 170-176.

A018. Boyer, K.E. & Zedler, J.B. (1999). Nitrogen addition could shift plant community composition in a restored California salt marsh. *Restor. Ecol.*, 7, 74-85.

A019. van Wijnen, H.J. & Bakker, J.P. (1999). Nitrogen and phosphorus limitation in a coastal barrier salt marsh: the implications for vegetation succession. *J. Ecol.*, 87, 265-272.

A020. Moon, D.C., Rossi, A.M. & Stiling, P. (2000). The effects of abiotically induced changes in host plant quality (and morphology) on a salt marsh planthopper and its parasitoid. *Ecol. Entomol.*, 25, 325-331.

A021. Moon, D.C. & Stiling, P. (2000). Relative importance of abiotically induced direct and indirect effects on a salt-marsh herbivore. *Ecology*, 81, 470-481.

A022. Boyer, K.E., Fong, P., Vance, R.R. & Ambrose, R.F. (2001). *Salicornia virginica* in a Southern California salt marsh: Seasonal patterns and a nutrient-enrichment experiment. *Wetlands*, 21, 315-326.

A023. Emery, N.C., Ewanchuk, P.J. & Bertness, M.D. (2001). Competition and salt-marsh plant zonation: Stress tolerators may be dominant competitors. *Ecology*, 82, 2471-2485.

A024. Silliman, B.R. & Zieman, J.C. (2001). Top-down control of *Spartina alterniflora* production by periwinkle grazing in a Virginia salt marsh. *Ecology*, 82, 2830-2845.

A025. Denno, R.F., Gratton, C., Peterson, M.A., Langellotto, G.A., Finke, D.L. & Huberty, A.F. (2002). Bottom-up forces mediate natural-enemy impact in a phytophagous insect community. *Ecology*, 83, 1443-1458.

A026. Moon, D.C. & Stiling, P. (2002). The effects of salinity and nutrients, on a tritrophic salt-marsh system. *Ecology*, 83, 2465-2476.

A027. Moon, D.C. & Stiling, P. (2002). The influence of species identity and herbivore feeding mode on top-down and bottom-up effects in a salt marsh system. *Oecologia*, 133, 243-253.

A028. Pennings, S.C., Stanton, L.E. & Brewer, J.S. (2002). Nutrient effects on the composition of salt marsh plant communities along the southern Atlantic and Gulf coasts of the United States. *Estuaries*, 25, 1164-1173.

A029. Denno, R.F., Gratton, C., Dobel, H. & Finke, D.L. (2003). Predation risk affects relative strength of top-down and bottom-up impacts on insect herbivores. *Ecology*, 84, 1032-1044.

A030. Feller, I.C., Whigham, D.F., McKee, K.L. & Lovelock, C.E. (2003). Nitrogen limitation of growth and nutrient dynamics in a disturbed mangrove forest, Indian River Lagoon, Florida. *Oecologia*, 134, 405-414.

A031. Feller, I.C., McKee, K.L., Whigham, D.F. & O'Neill, J.P. (2003). Nitrogen vs. phosphorus limitation across an ecotonal gradient in a mangrove forest. *Biogeochemistry*, 62, 145-175.

A032. Gratton, C. & Denno, R.F. (2003). Inter-year carryover effects of a nutrient pulse on *Spartina* plants, herbivores, and natural enemies. *Ecology*, 84, 2692-2707.

A033. Gratton, C. & Denno, R.F. (2003). Seasonal shift from bottom-up to top-down impact in phytophagous insect populations. *Oecologia*, 134, 487-495.

A034. Moon, D.C. & Stiling, P. (2003). The influence of legacy effects and recovery from perturbations in a tritrophic salt marsh complex. *Ecol. Entomol.*, 28, 457-466.

A035. Tessier, M., Vivier, J.P., Ouin, A., Gloaguen, J.C. & Lefeuvre, J.C. (2003). Vegetation dynamics and plant species interactions under grazed and ungrazed conditions in a western European salt marsh. *Acta Oecol.*, 24, 103-111.

A036. Tyler, A.C., Mastronicola, T.A. & McGlathery, K.J. (2003). Nitrogen fixation and nitrogen limitation of primary production along a natural marsh chronosequence. *Oecologia*, 136, 431-438.

A037. Lovelock, C.E., Feller, I.C., McKee, K.L., Engelbrecht, B.M.J. & Ball, M.C. (2004). The effect of nutrient enrichment on growth, photosynthesis and hydraulic conductance of dwarf mangroves in Panama. *Funct. Ecol.*, 18, 25-33.

A038. McFalls, T.B. (2004). Effects of disturbance and fertility upon the vegetation of a Louisiana coastal marsh. Southeastern Louisiana University

A039. Moon, D.C. & Stiling, P. (2004). The influence of a salinity and nutrient gradient on coastal vs. upland tritrophic complexes. *Ecology*, 85, 2709-2716.

A040. Ngai, J.T. & Jefferies, R.L. (2004). Nutrient limitation of plant growth and forage quality in Arctic coastal marshes. *J. Ecol.*, 92, 1001-1010.

A041. Wigand, C., Thursby, G.B., McKinney, R.A. & Santos, A.F. (2004). Response of *Spartina patens* to dissolved inorganic nutrient additions in the field. *J. Coast. Res.*, 134-149.

A042. Bos, D., Drent, R.H., Rubinigg, M. & Stahl, J. (2005). The relative importance of food biomass and quality for patch and habitat choice in Brent Geese Branta bernicla. *Ardea*, 93, 5-16.

A043. Huang, X.Q. & Morris, J.T. (2005). Distribution of phosphatase activity in marsh sediments along an estuarine salinity gradient. *Mar. Ecol. Progr. Ser.*, 292, 75-83.

A044. Hines, J., Lynch, M.E. & Denno, R.F. (2005). Sap-feeding insect communities as indicators of habitat fragmentation and nutrient subsidies. *J. Insect Conserv.*, 9, 261-280.

A045. Stiling, P. & Moon, D.C. (2005). Quality or quantity: the direct and indirect effects of host plants on herbivores and their natural enemies. *Oecologia*, 142, 413-420.

A046. Stiling, P. & Moon, D. (2005). Are trophodynamic models worth their salt? Top-down and bottom-up effects along a salinity gradient. *Ecology*, 86, 1730-1736.

A047. Traut, B.H. (2005). Effects of nitrogen addition and salt grass (*Distichlis spicata*) upon high salt marsh vegetation in northern California, USA. *Estuaries*, 28, 286-295.

A048. Albarracin, M.T. & Stiling, P. (2006). Bottom-up and top-down effects on insect herbivores do not vary among sites of different salinity. *Ecology*, 87, 2673-2679.

A049. Hines, J., Megonigal, J.P. & Denno, R.F. (2006). Nutrient subsidies to belowground microbes impact aboveground food web interactions. *Ecology*, 87, 1542-1555.

A050. Huberty, A.F. & Denno, R.F. (2006). Consequences of nitrogen and phosphorus limitation for the performance of two planthoppers with divergent life-history strategies. *Oecologia*, 149, 444-455.

A051. Lovelock, C.E., Ball, M.C., Feller, I.C., Engelbrecht, B.M.J. & Ewe, M.L. (2006). Variation in hydraulic conductivity of mangroves: influence of species, salinity, and nitrogen and phosphorus availability. *Physiol. Plantarum*, 127, 457-464.

A052. van der Graaf, A.J., Stahl, J., Veen, G.F., Havinga, R.M. & Drent, R.H. (2007). Patch choice of avian herbivores along a migration trajectory-from temperate to arctic. *Basic Appl. Ecol.*, 8, 354-363.

A053. Crain, C.M. (2007). Shifting nutrient limitation and eutrophication effects in marsh vegetation across estuarine salinity gradients. *Estuar. Coast.*, 30, 26-34.

A054. Feller, I.C. & Chamberlain, A. (2007). Herbivore responses to nutrient enrichment and landscape heterogeneity in a mangrove ecosystem. *Oecologia*, 153, 607-616.

A055. Feller, I.C., Lovelock, C.E. & McKee, K.L. (2007). Nutrient addition differentially affects ecological processes of *Avicennia germinans* in nitrogen versus phosphorus limited mangrove ecosystems. *Ecosystems*, 10, 347-359.

A056. Lovelock, C.E., Feller, I.C., Ellis, J., Schwarz, A.M., Hancock, N., Nichols, P. et al. (2007). Mangrove growth in New Zealand estuaries: the role of nutrient enrichment at sites with contrasting rates of sedimentation. *Oecologia*, 153, 633-641.

A057. Tyler, A.C., Lambrinos, J.G. & Grosholz, E.D. (2007). Nitrogen inputs promote the spread of an invasive marsh grass. *Ecol. Appl.*, 17, 1886-1898.

A058. Bertness, M.D., Crain, C., Holdredge, C. & Sala, N. (2008). Eutrophication and consumer control of New England salt marsh primary productivity. *Conserv. Biol.*, 22, 131-139.

A059. Sala, N.M., Bertness, M.D. & Silliman, B.R. (2008). The dynamics of bottom-up and top-down control in a New England salt marsh. *Oikos*, 117, 1050-1056.

A060. McFarlin, C.R., Brewer, J.S., Buck, T.L. & Pennings, S.C. (2008). Impact of fertilization on a salt marsh food web in Georgia. *Estuar. Coast.*, 31, 313-325.

A061. Daleo, P., Alberti, J., Canepuccia, A., Escapa, M., Fanjul, E., Silliman, B.R. et al. (2008). Mycorrhizal fungi determine salt-marsh plant zonation depending on nutrient supply. *J. Ecol.*, 96, 431-437.

A062. Pennings, S.C. & Simpson, J.C. (2008). Like herbivores, parasitic plants are limited by host nitrogen content. *Plant Ecol.*, 196, 245-250.

A063. Bertness, M.D., Holdredge, C. & Altieri, A.H. (2009). Substrate mediates consumer control of salt marsh cordgrass on Cape Cod, New England. *Ecology*, 90, 2108-2117.

A064. Hargreaves, S.K., Horrigan, E.J. & Jefferies, R.L. (2009). Seasonal partitioning of resource use and constraints on the growth of soil microbes and a forage grass in a grazed Arctic salt-marsh. *Plant Soil*, 322, 279-291.

A065. Whigham, D.F., Verhoeven, J.T.A., Samarkin, V. & Megonigal, P.J. (2009). Responses of *Avicennia germinans* (black mangrove) and the soil microbial community to nitrogen addition in a hypersaline wetland. *Estuar. Coast.*, 32, 926-936.

A066. Alberti, J., Casariego, A.M., Daleo, P., Fanjul, E., Silliman, B., Bertness, M. et al. (2010). Abiotic stress mediates top-down and bottom-up control in a Southwestern Atlantic salt marsh. *Oecologia*, 163, 181-191.

A067. Graham, S.A. & Mendelssohn, I.A. (2010). Multiple levels of nitrogen applied to an oligohaline marsh identify a plant community response sequence to eutrophication. *Mar. Ecol. Progr. Ser.*, 417, 73-82.

A068. Martin, K.C., Bruhn, D., Lovelock, C.E., Feller, I.C., Evans, J.R. & Ball, M.C. (2010). Nitrogen fertilization enhances water-use efficiency in a saline environment. *Plant Cell Environ.*, 33, 344-357.

A069. Reef, R., Ball, M.C., Feller, I.C. & Lovelock, C.E. (2010). Relationships among RNA : DNA ratio, growth and elemental stoichiometry in mangrove trees. *Funct. Ecol.*, 24, 1064-1072.

A070. Wimp, G.M., Murphy, S.M., Finke, D.L., Huberty, A.F. & Denno, R.F. (2010). Increased primary production shifts the structure and composition of a terrestrial arthropod community. *Ecology*, 91, 3303-3311.

A071. Alberti, J., Cebrian, J., Casariego, A.M., Canepuccia, A., Escapa, M. & Iribarne, O. (2011). Effects of nutrient enrichment and crab herbivory on a SW Atlantic salt marsh productivity. *J. Exper. Mar. Biol. Ecol.*, 405, 99-104.

A072. Alberti, J., Canepuccia, A., Pascual, J., Perez, C. & Iribarne, O. (2011). Joint control by rodent herbivory and nutrient availability of plant diversity in a salt marsh-salty steppe transition zone. *J. Veg. Sci.*, 22, 216-224.

A073. Lovelock, C.E., Feller, I.C., Adame, M.F., Reef, R., Penrose, H.M., Wei, L.L. et al. (2011). Intense storms and the delivery of materials that relieve nutrient limitations in mangroves of an arid zone estuary. *Funct. Plant Biol.*, 38, 514-522.

A074. Nelson, J.L. & Zavaleta, E.S. (2012). Salt marsh as a coastal filter for the oceans: changes in function with experimental increases in nitrogen loading and sea-level rise. *PLoS ONE*, 7, e38558.

A075. Ryan, A.B. & Boyer, K.E. (2012). Nitrogen further promotes a dominant salt marsh plant in an increasingly saline environment. *J. Plant Ecol.*, 5, 429-441.

A076. McCall, B.D. & Pennings, S.C. (2012). Geographic variation in salt marsh structure and function. *Oecologia*, 170, 777-787.

A077. Reef, R., Ball, M.C. & Lovelock, C.E. (2012). The impact of a locust plague on mangroves of the arid Western Australia coast. *J. Trop. Ecol.*, 28, 307-311.

A078. Feller, I.C., Chamberlain, A.H., Piou, C., Chapman, S. & Lovelock, C.E. (2013). Latitudinal patterns of herbivory in mangrove forests: consequences of nutrient over-enrichment. *Ecosystems*, 16, 1203-1215.

A079. Sparks, E. & Cebrian, J. (2014). Effects of fertilization on grasshopper grazing of northern Gulf of Mexico salt marshes. *Estuar. Coast.*, doi:10.1007/s12237-014-9858-6

A080. Vivanco, L., Irvine, I.C. & Martiny, J.B.H. (2015). Nonlinear responses in salt marsh functioning to increased nitrogen addition. *Ecology*, doi:10.1890/13-1983.1

| **Variable** | **df** | **T** | **P** |
| --- | --- | --- | --- |
| Belowground Biomass (g/m2) | 3.9 | -0.83 | 0.45 |
| Number Flowering/ m2 | 3.2 | 1.3 | 0.27 |
| August New Shoots/ m2 | 3.2 | 0.5 | 0.65 |
| September New Shoots/ m2 | 2.0 | -0.86 | 0.47 |
| August Mean Length of New Shoots (cm) | 2.7 | -2.3 | 0.11 |
| September Mean Length of New Shoots (cm) | 2.2 | -0.72 | 0.54 |

**S2 Table:** **T-test results between cage and cage control plots for six variables.**

**S3 Table:** **Tukey post-hoc test determining the significant difference between means (n=3) of dependent variables.**

| **Variable** | **Sources** | **Compared Treatments** | **Difference in**  **Means** | ***P*** |
| --- | --- | --- | --- | --- |
| Belowground Biomass (g/m^2^) | **Grazer level**  **Nitrogen** | Removal-Addition  Fertilized-Ambient | 114.1  77.4 | 0.027  0.028 |
| Ratio Below: Aboveground Biomass (g/m^2^) | **Grazer Level**  **Nitrogen** | Addition- Removal  Addition- Control  Fertilized: Ambient | 0.453  0.406  0.448 | <0.001  <0.001  <0.001 |
| Radulations (cm/stem) | **Grazer Level** | Addition-Removal | 1.10 | 0.010 |
| Standing Dead Mass (g/m^2^) | **Interaction**  Addition: Fertilized - Control: Ambient Control: Fertilized - Control: Ambient  Addition: Fertilized - Removal: Fertilized Control: Fertilized - Removal: Fertilized  Addition: Fertilized - Removal: Ambient Control: Fertilized - Removal: Ambient  Addition: Fertilized - Addition: Ambient Control: Fertilized - Addition: Ambient  Control: Fertilized - Addition: Fertilized  **Grazer Level**  Addition- Removal  Control- Removal  **Nitrogen**  Fertilized: Ambient | |  |  |
|  |  |  | 7.67 | 0.0021 |
|  |  |  | 15.24 | <0.001 |
|  |  |  | 6.58 | 0.0072 |
|  |  |  | 14.15 | <0.001 |
|  |  |  | 5.67 | 0.021 |
|  |  |  | 13.24 | <0.001 |
|  |  |  | 4.88 | 0.051 |
|  |  |  | 12.45 | <0.001 |
|  |  |  | 7.57 | 0.002 |
|  |  |  | 3.69 | 0.010 |
|  |  |  | 6.07 | <0.001 |
|  |  |  | 6.4 | <0.001 |
| Leaf Litter (g/m^2^) |  | |  |  |
|  | **Interaction** | |  |  |
|  | Addition: Fertilized - Control: Ambient | | 0.33 | 0.090 |
|  | Removal: Ambient - Control: Ambient | | 0.64 | 0.001 |
|  | Removal: Ambient - Removal: Fertilized | | 0.59 | 0.002 |
|  | Removal: Ambient - Addition: Ambient | | 0.55 | 0.003 |
|  | Removal: Ambient - Control: Fertilized | | 0.33 | 0.090 |
|  | **Grazer Level** | |  |  |
|  | Removal- Control | | 0.19 | 0.077 |
| Number Flowering/ m^2^ | **Interaction** | |  |  |
|  | Control: Nitrogen - Addition: Ambient | | 18 | 0.054 |
|  | Removal: Ambient - Addition: Ambient | | 22.67 | 0.013 |
|  | Removal: Nitrogen - Addition: Ambient | | 53.33 | <0.001 |
|  | Removal: Ambient - Addition: Nitrogen | | 19 | 0.040 |
|  | Removal: Nitrogen - Addition: Nitrogen | | 49.67 | <0.001 |
|  | Removal: Nitrogen - Control: Ambient | | 46 | <0.001 |
|  | Removal: Nitrogen - Removal: Ambient | | 30.67 | 0.001 |
|  | **Grazer Level** | |  |  |
|  | Control-Addition | | 10.83 | 0.039 |
|  | Removal-Addition | | 36.17 | <0.001 |
|  | Removal-Control | | 25.33 | <0.001 |
|  | **Nitrogen** | |  |  |
|  | Nitrogen-Ambient | | 15 | <0.001 |
| Proportion Flowering/ m^2^ |  | |  |  |
|  | **Grazer Level** | |  |  |
|  | Control-Addition | | 0.037 | 0.03 |
|  | Removal-Addition | | 0.11 | <0.001 |
|  | Removal-Control | | 0.08 | <0.001 |
|  | **Nitrogen** | |  |  |
|  | Fertilized-Ambient | | 0.04 | 0.002 |
| August New Shoots/ m^2^ |  | |  |  |
|  | **Grazer Level** | |  |  |
|  | Removal-Addition | | 117.33 | 0.050 |
|  | Removal-Control | | 138.67 | 0.020 |
|  | **Nitrogen** | |  |  |
|  | Fertilized-Ambient | | 140.44 | 0.002 |
| September New Shoots/ m^2^ | **Grazer Level** | Removal-Addition  Removal-Control | 221.33  157.33 | 0.010  0.070 |
| August Mean Length of New  Shoots (cm) | **Nitrogen** | Fertilized-Ambient | 3.03 | <0.001 |
| September Mean Length of  New Shoots (cm) | **Nitrogen** | Fertilized-Ambient | 3.36 | 0.001 |

Only the significant differences reported in table 1 are reported here.
